# Syntheses of Specifically ^15^N‐Labeled Adenosine and Guanosine

**DOI:** 10.1002/cpz1.612

**Published:** 2022-12-19

**Authors:** Roger A. Jones, Barbara L. Gaffney

**Affiliations:** ^1^ Chemistry and Chemical Biology Rutgers University New Brunswick New Brunswick New Jersey

**Keywords:** carbon‐13 labeled nucleosides, labeled nucleosides, nitrogen‐15 labeled nucleosides, ^15^N‐labeled adenosine, ^15^N‐labeled guanosine

## Abstract

This article describes the specific incorporation of ^15^N into the N7 and amino positions of adenosine (Basic Protocol 1), and conversion of the adenosine to guanosine labeled at the N1, N7, and amino positions (Basic Protocol 2). Two variations of the procedures are also presented that include either ^12^C or ^13^C at the C8 position of adenosine, and ^13^C at either the C8 or C2 position of guanosine. These ^13^C tags permit the incorporation of two ^15^N‐labeled nucleosides into an RNA strand while ensuring that their nuclear magnetic resonance (NMR) signals can be distinguished from each other by the presence or absence of C‐N coupling. While the major application of these specifically ^15^N‐labeled nucleosides is NMR, the additional mass makes them useful in mass spectrometry (MS) as well. The procedures can also be adapted to synthesize the labeled deoxynucleosides. The Support Protocol describes the synthesis of 7‐methylguanosine. © 2022 The Authors. Current Protocols published by Wiley Periodicals LLC.

**Basic Protocol 1**: Syntheses of [7,NH_2_‐^15^N_2_]‐ and [8‐^13^C‐7,NH_2_‐^15^N_2_]adenosine

**Support Protocol**: Synthesis of 7‐methylguanosine

**Basic Protocol 2**: Synthesis of [2‐^13^C‐1,7,NH_2_‐^15^N_3_]‐ and [8‐^13^C‐1,7,NH_2_‐^15^N_3_]guanosine

## Basic Protocol 1 SYNTHESES OF [7,NH_2_‐^15^N_2_]‐ AND [8‐^13^C‐7,NH_2_‐^15^N_2_]ADENOSINE

**Figure 1:**
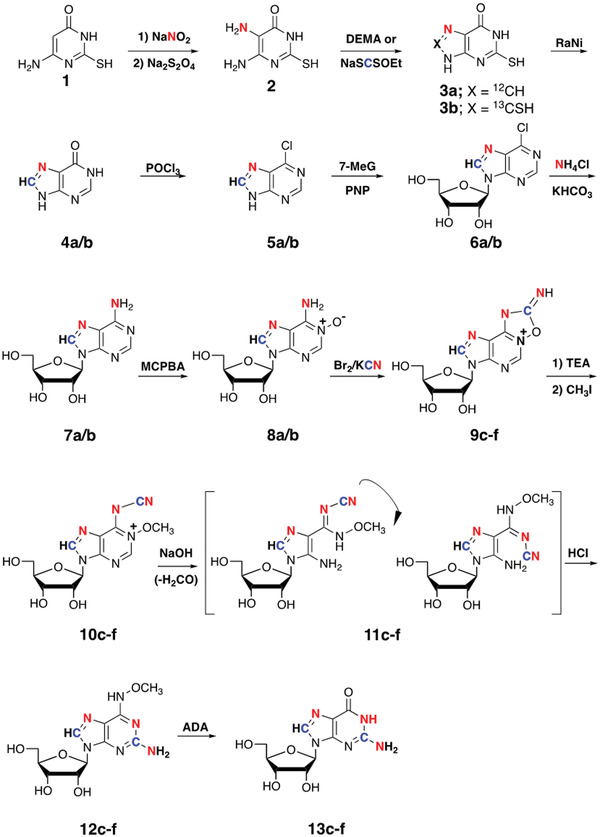
Steps for synthesis of [7,NH_2_‐^15^N_2_]‐ and [8‐^13^C‐7,NH_2_‐^15^N_2_]adenosine using Basic Protocol 1, and for synthesis of [2‐^13^C‐1,7,NH_2_‐^15^N_3_]‐ and [8‐^13^C‐1,7,NH_2_‐^15^N_3_]guanosine using Basic Protocol 2. In the figure, nitrogen atoms in red indicate ^15^N while carbon atoms in blue indicate that either ^12^C or ^13^C may be incorporated. The specific designations are: (1) ^12^C8, (2) ^13^C8, (3) ^12^C8 and ^12^C2, (4) ^12^C8 and ^13^C2, and (5) ^13^C8 and ^12^C2, (6) ^13^C8 and ^13^C2. Abbreviations: DEMA, diethoxymethyl acetate; 7‐MeG, 7‐methylguanosine; PNP, purine nucleoside phosphorylase; RaNi, Raney nickel; ADA, adenosine deaminase.

As shown in Figure 1, the procedures described here (Pagano, Lajewski, & Jones, 1995; Shallop & Jones, 2000; Zhao et al., 1997) start with the inexpensive pyrimidine 4‐amino‐6‐hydroxy‐2‐mercaptopyrimidine (**1**). The first ^15^N label is introduced by a direct nitrosation/reduction to give **2**. This is followed by ring closure using either diethoxymethyl acetate in dimethylformamide (DMF) to give **3a** with a ^12^C at the C8 position, or [^13^C]sodium ethyl xanthate to give **3b** with a ^13^CSH at the C8 position. Removing the thiol group(s) with Raney nickel forms hypoxanthine (**4a/b**), which can readily be converted to 6‐chloropurine (**5a/b**), which are excellent substrates for enzymatic transglycosylation. The second ^15^N label is then introduced into the nucleoside by displacement of the chloride by ^15^NH_3_, which is generated in situ to give the labeled adenosines **7a/b**.

*CAUTION*: The procedures in this article use a number of highly toxic and dangerous reagents. Raney nickel is pyrophoric when dry and may burst into flames if not kept wet. Phosphorous oxychloride (POCl_3_) is very reactive and hydrolyzes to hydrochloric and phosphoric acids, which are both highly corrosive to skin and tissue. Cyanogen bromide is very dangerous; improper use of this reagent can cause death. All reactions must be done with great care in an appropriate chemical fume hood.

### Materials

4‐Amino‐6‐hydroxy‐2‐mercaptopyrimidine monohydrate (**1**; also called 6‐amino‐2‐thioxo‐1,2‐dihydro‐4(3*H*)‐pyrimidinone; MilliporeSigma)
1 N HCl
[^15^N]Sodium nitrite ([^15^N]NaNO_2_; Isotec or Cambridge Isotope Laboratories)
2:98 to 40:60 (v/v) gradient of acetonitrile/0.1 M triethylammonium acetate (TEAA), pH 6.8
95% (v/v) ethanol, 4°C
Acetone, 4°C
Phosphorous pentoxide (P_2_O_5_)
Saturated aqueous NaHCO_3_
Sodium hydrosulfite (Na_2_S_2_O_4_)
Glacial acetic acid
96% (v/v) formic acid
Nitrogen gas source
Dimethylformamide (DMF), anhydrous
Diethoxymethyl acetate (DEMA), for ^12^C synthesis only
Acetonitrile, room temperature and 4°C
[^13^C]Sodium ethyl xanthate ([^13^C]NaSCSOEt; see recipe), for ^13^C synthesis only
NaOH
50% aqueous Raney 2800 nickel (RaNi) slurry (MilliporeSigma)
Dipotassium salt of EDTA
Phosphorous oxychloride (POCl_3_)
*N,N*‐Dimethylaniline
5% (v/v) NH_3_ (diluted with water from 30% concentrated aqueous ammonia)
Ethyl acetate
Ethyl ether
1 M HCl
7‐Methylguanosine (see Support Protocol)
0.02 M K_2_HPO_4_
3 M NaOH
Purine nucleoside phosphorylase (MilliporeSigma)
[^15^N]Ammonium chloride ([^15^N]NH_4_Cl; Isotec or Cambridge Isotope Laboratories)
Dimethylsulfoxide (DMSO), anhydrous
KHCO_3_, anhydrous

100‐ and 250‐ml round‐bottom flasks
Small glass vials
1‐, 3‐, 10‐, and 20‐ml syringes
Vacuum desiccator
Rubber septa fitted with large‐bore vent needles
Condenser
Rotary evaporator, connected to water aspirator and a vacuum pump, the latter with a dry‐ice trap
Oil bath (silicone oil), 130°C
Separatory funnel
Continuous extraction apparatus for solvents lighter than water (MilliporeSigma)
30°C oven with shaker
50‐ml bomb with Teflon liner (Parr Instrument)
80°C oven

Additional reagents and equipment for analytical and preparative reversed‐phase high‐performance liquid chromatography (HPLC; Current Protocols article: Sinha & Jung, 2015)

### Synthesize [5‐^15^N]‐5,6‐diamino‐2‐thioxo‐1,2‐dihydro‐4(3H)‐pyrimidinone (2)

1 Weigh 0.805 g (5.0 mmol) 4‐amino‐6‐hydroxy‐2‐mercaptopyrimidine monohydrate (**1**) into a 100‐ml round‐bottom flask with a stir bar.
2 Add 25 ml of 1 N HCl and chill suspension 10 min in an ice bath.
3 Weigh 0.385 g [5.5 mmol, 1.1 equivalents (eq)] [^15^N]NaNO_2_ into a small glass vial, dissolve in ∼1 ml water, and draw solution into a 3‐ml syringe.
4 Slowly add the sodium nitrite to the reaction mixture over ∼5 min.
  If the addition is too fast, the reaction may bubble over. Before long, the mixture will change from yellow to red.
5 Stir mixture ∼7 hr in the ice bath, while monitoring the reaction for completeness by reversed‐phase HPLC with a gradient of 2:98 to 40:60 acetonitrile/0.1 M TEAA over 5 min.
  The peak representing the starting material should diminish to <3% and a new peak representing the product should appear.
  Any reversed‐phase column can be used. The sample should be injected immediately after mixing for comparison.
6 Collect solid by vacuum filtration and wash it with 5 ml cold water, then 5 ml cold 95% ethanol, and finally 5 ml cold acetone.
7 Without removing it from the funnel, dry over P_2_O_5_ in a vacuum desiccator overnight.
  The yield is usually >95%. See Table 1 for various data on this and other intermediates and products.

**Table 1:** Molecular Weights, TLC and HPLC Mobilities, and UV λ_max_ of ^15^N‐Labeled Adenosine and Guanosine Intermediates**^*a*^**

Compound	Mol. wt. (Da)	TLC *R*_f_ *^b^*	HPLC retention time (min)*^c^*	UV λ_max_ (nm)
**2**	159	0.0	0.8	301
**3a**	169	0.2	0.7	280
**3b**	202	0.0	1.4	299
**4a**	137	0.1	0.8	250
**4b**	138	0.1	0.8	250
**5a**	155	0.3	2.4	265
**5b**	156	0.3	2.4	265
**6a**	287	0.2	4.5	264
**6b**	288	0.2	4.5	264
**7a**	269	0.1	2.6	259
**7b**	270	0.1	2.6	259
**8a**	285	0.0	0.9	295
**8b**	286	0.0	0.9	295
**12c**	315	0.1	3.3	280
**12d**	316	0.1	3.3	280
**12e**	316	0.1	3.3	280
**12f**	317	0.1	3.3	280
**13c**	286	0.0	1.4	253
**13d**	287	0.0	1.4	253
**13e**	287	0.0	1.4	253
**13f**	288	0.0	1.4	253

^*a*^Abbreviations: HPLC, high‐performance liquid chromatography; Mol. wt., molecular weight; TLC, thin‐layer chromatography.

^*b*^ *R*_f_ values determined with 10:90 (v/v) CH_3_OH/CH_2_Cl_2_.

^*c*^Gradient of 2:98 to 40:60 (v/v) acetonitrile/0.1 M triethylammonium acetate, pH 6.8, over 5 min, on a Waters NovaPak or Atlantis C18 column.

8 Scrape out most of the solid from the funnel into a 100‐ml round‐bottom flask. Rinse funnel with portions of saturated aqueous NaHCO_3_ and add them to the flask to give a final volume of 40 ml.
9 Add a stir bar and place mixture in an ice bath for 10 min. Stir gently.
10 Quickly weigh 2.61 g (15 mmol, 3 eq) Na_2_S_2_O_4_ into a small beaker. Using a spatula, gradually add it in portions to the reaction over 20 min.
  The sodium hydrosulfite has a strong odor and should be kept in the back of the hood. If the addition is too fast, the reaction will bubble over.
11 Insert a rubber septum containing a large‐bore vent needle.
12 Stir mixture ∼7 hr in the ice bath, while monitoring the reaction for completeness by HPLC as described (step 5).
  The mixture will change from red to yellow.
13 Slowly add 1.6 ml glacial acetic acid over ∼5 min to neutralize the NaHCO_3_ and stir another 5 min.
14 Collect product by vacuum filtration and wash twice with 5 ml cold water and then twice with 5 ml cold 95% ethanol.
15 Without removing it from the funnel, dry **2** over P_2_O_5_ in a vacuum desiccator overnight.
  The yield is usually >95%.
  The synthesis is continued using either diethoxymethyl acetate in DMF to give ^12^C at the 8 position (steps 16a to 26a) or [^13^C]sodium ethyl xanthate to give ^13^C at the 8 position (steps 16b to 22b).

### Perform ring closure

#### For [7‐^15^N]‐2‐thioxohypoxanthine (**3a**)

16a Scrape out most of **2** from the funnel into a 100‐ml round‐bottom flask. Rinse funnel with portions of 96% formic acid and add them to the flask to give a final volume of 25 ml.
17a Add a stir bar, attach a condenser, and reflux the solution 1 hr to make the formate salt.
18a Concentrate to dryness using a rotary evaporator and scrape down the sides of the flask with a spatula, if necessary.
19a Insert a rubber septum and displace the air with nitrogen.
20a Use syringes to add the following through the septum:
  20 ml anhydrous DMF;
  1.63 ml DEMA (10 mmol, 2 eq);
  0.24 ml of 96% formic acid (6 mmol, 1.2 eq).
21a Heat mixture 3 hr in an oil bath set at 130°C. Follow reaction by HPLC.
  The flask is lifted from the oil bath and allowed to cool briefly, and then a small syringe with a long, dry needle is used to get a sample for HPLC.
22a Cool flask, concentrate solution to a solid using a rotary evaporator, and loosen it with a spatula if necessary.
23a Add 15 ml acetonitrile to the flask, attach a condenser, and reflux 10 min using the 130°C oil bath.
24a Cool flask to room temperature, add 10 ml acetonitrile, and then chill in an ice bath.
25a Collect **3a** by vacuum filtration and wash twice with 5 ml cold acetonitrile.
26a Without removing it from the funnel, dry **3a** over P_2_O_5_ in the vacuum desiccator overnight. Proceed to step 27.
  The yield is usually >95%.

#### For [8‐^13^C‐7‐^15^N]‐2,8‐dithioxohypoxanthine (**3b**)

16b Scrape out most of **2** from the funnel into a 100‐ml round‐bottom flask and add 0.80 g (5.5 mmol) [^13^C]NaSCSOEt.
17b Insert a condenser into the flask, attach a nitrogen line and vent needle, and displace air for 5 min.
18b Add 15 ml DMF and reflux mixture under nitrogen ∼3 hr, using HPLC to monitor the reaction.
19b Cool mixture in an ice bath and add 50 ml cold acetonitrile to precipitate **3b**.
20b Collect the solid **3b** by vacuum filtration and wash twice with 5 ml cold acetonitrile. Save filtrate and both washes.
21b Concentrate filtrate and washes, and purify by preparative reversed‐phase chromatography.
22b Dry the combined portions of **3b** over P_2_O_5_ in a vacuum desiccator overnight. Continue with step 27.
  The yield is usually >95%.

### Synthesize [7‐^15^N]hypoxanthine and [8‐^13^C‐7‐^15^N]hypoxanthine (4a/b)

27 Scrape out most of **3a/b** from the funnel into a 100‐ml round‐bottom flask.
28 Rinse funnel with portions of water and add them to the flask to give a final volume of 30 ml.
29 Add a stir bar and 2 ml of 96% formic acid.
30 Weigh 4.5 g of 50% aqueous RaNi slurry into a small glass vial or beaker.
  CAUTION: RaNi is pyrophoric (will spontaneously burst into flames) if it is allowed to dry out. To weigh it, shake the bottle and immediately transfer some of the suspended RaNi to the vial. Continue adding the suspension (shaking the bottle each time) until 4.5 g of the 50:50 mixture of RaNi/water has been measured out.
31 Using a dropper, transfer the RaNi suspension over 5 min to the reaction flask and add 1.5 g dipotassium salt of EDTA.
  Traces of remaining RaNi in the vial and dropper should be destroyed with 6 M HCl.
32 Connect a condenser to the flask. Using the 130°C oil bath, reflux ∼2 hr while monitoring the reaction for completeness by HPLC.
33 Remove flask from the oil bath and allow it to cool only briefly.
34 Remove condenser from the flask and carefully filter the hot reaction mixture to remove the RaNi. Rinse the flask and then the funnel with three 10‐ml portions of boiling water, adding these washes to the filtrate.
  To destroy the RaNi remaining in the funnel, the funnel should be transferred to a large beaker and portions of 6 M HCl should be slowly added until no black particles can be observed.
35 Concentrate filtrate and washes to dryness in a 100‐ml round‐bottom flask using the rotary evaporator.
36 Dry **4a/b** over P_2_O_5_ in the vacuum desiccator overnight.
  The yield is usually >95%. **4a/b** can be purified by reversed‐phase chromatography if desired.

### Synthesize [7‐^15^N]‐ and [8‐^13^C‐7‐^15^N]‐6‐chloropurine (5a/b)

37 Weigh 0.69 g (5.0 mmol) **4a/b** into a very dry 100‐ml round‐bottom flask.
38 Add 20 ml (215 mmol, 43 eq) POCl_3_ and 2 ml (16 mmol) *N,N*‐dimethylaniline.
  Use great care with POCl_3_; it is very reactive.
39 Attach a condenser and reflux 20 min under nitrogen.
  The resulting solution should be black and homogeneous. It is essential for this reaction to remain anhydrous.
40 Monitor reaction for completeness by HPLC and continue refluxing the mixture for ≤30 min more.
41 Concentrate mixture to a very small volume using a rotary evaporator, first with an aspirator and then with a vacuum pump protected with a dry‐ice trap. Add 10 ml *N,N*‐dimethylaniline and continue the evaporation.
  It is necessary to remove all traces of POCl_3_, or localized heating from later neutralization with NH_3_ may cause the reaction to reverse. Be extremely careful to dry the evaporator condenser and trap prior to use, to keep dry ice in the traps throughout this process, and then to pour the collected POCl_3_ into a plastic container of ice to decompose it.
42 Cool flask in an ice bath and very slowly add 30 ml of 5% NH_3_ to dissolve the black gum.
  As a neutral compound, 6‐chloropurine is insoluble in water but under basic conditions it will ionize and therefore dissolve.
43 Make sure the pH of the solution is >10 (add more NH_3_ if necessary) and then pour it into a separatory funnel. Wash it first with 30 ml ethyl acetate and then twice with 30 ml ethyl ether. Check the layers by HPLC.
44 Combine all organic layers that contain traces of product in a separatory funnel, backwash with 5% NH_3_, and add this aqueous layer to the main reaction mixture.
45 Concentrate this aqueous solution to dryness to remove all the NH_3_.
  It is necessary to remove all traces of NH_3_, or localized heating from later neutralization with HCl may cause the reaction to reverse. As the NH_3_ evaporates, it is very likely to “bump,” so a large enough flask should be used.
46 Add 20 ml water and chill the flask in an ice bath. Slowly acidify the solution to pH 2 using 1 M HCl.
  The mixture will turn cloudy.
47 Set up a continuous extraction apparatus for solvents lighter than water. Pour the aqueous layer into the extractor and add ethyl ether until the level is just under the side arm.
48 Fill a 250‐ml round‐bottom flask with ethyl ether, add a stir bar, connect to the extractor, and place it in an oil bath. Attach a condenser to the top of the extractor and start heating the oil bath to 45°C.
49 Continue the extraction for 3 to 4 days, using fresh ether each day. Check both the aqueous and ether layers each day by HPLC. Verify that the pH of the aqueous layer is still <2 and, if it is not, adjust it with 1 M HCl.
50 Concentrate the ether layers to a small volume, whereupon a significant amount of **5a/b** should crystallize out. Collect **5a/b** by vacuum filtration and check it for purity by HPLC. Save the filtrate.
51 Concentrate filtrate to dryness and dissolve it in 10 ml water. Purify by reversed‐phase preparative chromatography.
52 Concentrate fractions containing pure product to dryness. Dry both portions of **5a/b** in a vacuum desiccator over P_2_O_5_ overnight.
  The yield is usually between 80% and 90%.

### Synthesize [7‐^15^N]‐ and [8‐^13^C‐7‐^15^N]‐6‐chloro‐9‐(β‐d‐erythropentofuranosyl) purine (6a/b)

53 Place 2.12 g (7.5 mmol, 1.5 eq) 7‐methylguanosine and 0.78 g (5 mmol) **5a/b** into a 100‐ml round‐bottom flask and add 20 ml of 0.02 M K_2_HPO_4_. Using pH paper, adjust the pH to 7.4 with 3 M NaOH, if necessary.
54 Add 250 units of purine nucleoside phosphorylase. Insert a septum and heat the mixture ∼3 days in an oven at 30°C with gentle agitation. Monitor reaction for completeness each day by HPLC.
55 Pour mixture into 10 ml DMF and stir ∼1 hr at room temperature.
56 Filter suspension to remove most of the solid 7‐methylguanine. Suspend solid in 5 ml fresh DMF, stir 15 min, and filter.
57 Concentrate combined filtrates to a small volume using a rotary evaporator and add 15 ml water (more if necessary to dissolve the solid).
58 Purify the crude **6a/b** by preparative reversed‐phase HPLC and dry it over P_2_O_5_ in the vacuum desiccator overnight.
  The yield is usually 85% to 95%.

### Synthesize [7,NH_2_‐^15^N_2_]‐ and [8‐^13^C‐7,NH_2_‐^15^N_2_]adenosine (7a/b)

59 Place 1.44 g (5 mmol) **6a/b** into a clean Teflon liner of a bomb and add 0.54 g (10 mmol, 2 eq) of [^15^N]NH_4_Cl and 7 ml anhydrous DMSO.
60 Add 1.5 g (15 mmol, 3 eq) KHCO_3_ and immediately seal the bomb.
61 Heat the bomb 3 days in an 80°C oven, swirling the mixture once or twice each day.
62 Cool the bomb to room temperature and then to –20°C for ≥30 min. Open it carefully and dilute mixture with 10 ml water. Adjust the pH to 7 with glacial acetic acid.
  Care should be taken when opening the bomb because CO_2_ will have been generated.
63 Check the reaction by HPLC.
64 Purify **7a/b** by preparative reversed‐phase HPLC and dry over P_2_O_5_ in the vacuum desiccator overnight.
  The yield is usually 80% to 90%. See Tables 2 and 3 for NMR chemical shifts and coupling constants.

**Table 2:** Nuclear Magnetic Resonance Chemical Shifts (ppm) for ^15^N‐Labeled Adenosine (**7**) and Guanosine (**13**)^*a*^

Compound	N1**H**	H8	H2	N**H**_2_	1′H	2′H	3′H	4′H	5′H	N1	N7	**N**H_2_	C2	C8
**7a/b**	—	8.35	8.14	7.40	5.87	4.60	4.14	3.96	3.6	—	242	83	152	140
**13c‐f**	10.6	7.92	NA	6.44	5.68	4.38	4.07	3.85	3.6	148	244	75	154	135

^*a*^All NMR samples in DMSO‐*d_6_*. ^15^N data are relative to ^15^NH_3_ using external 1 M [^15^N]urea in DMSO at 77.0 ppm as a reference. Additional data (e.g., for intermediates) are available in Pagano et al. (1995), Zhao et al. (1997), and Shallop and Jones (2000). The bold indicates the atoms (H, N) to which the ppm chemical shift refers.

**Table 3:** Nuclear Magnetic Resonance Coupling Constants (Hz) for ^15^N‐Labeled Adenosine (**7**) and Guanosine (**13**)

Compound	^13^C8‐^15^N7	^13^C2‐^15^N1	^13^C2‐^15^NH_2_
**7b**	<1	NA	NA
**13d/f**	<1	12	24

## SYNTHESIS OF 7‐METHYLGUANOSINE

Although 7‐methylguanosine can be purchased from MilliporeSigma, it is quite easy to make. Commercial 7‐methylguanosine is very expensive and the purity may not be as high.

### Materials

Guanosine
*N,N*‐Dimethylacetamide
Nitrogen gas source
Dimethyl sulfate
Concentrated aqueous NH_3_
Acetone, 4°C
95% (v/v) ethanol
Ethyl ether

250‐ml round‐bottom flask
Rubber septum
10‐ml syringes

*CAUTION*: Dimethyl sulfate is very dangerous because it is a potent alkylating agent; wear gloves and use caution.

### Synthesize 7‐methylguanosine

1 Place 9.91 g (35 mmol) guanosine in a 250‐ml round‐bottom flask along with a stir bar.
2 Add 80 ml *N,N*‐dimethylacetamide as a solvent, insert a rubber septum, and displace the air with nitrogen gas.
3 Carefully withdraw 7.0 ml (74 mmol, 2.1 eq) dimethyl sulfate with a 10‐ml syringe and add it to the suspension.
4 Stir mixture ∼6 hr and monitor reaction for completeness by HPLC.
5 Slowly add 10 ml concentrated aqueous NH_3_ and then check the pH using pH paper. Continue to add more NH_3_ slowly, frequently checking the pH, until the mixture is pH 10.
  This step is essential and quenches the excess dimethyl sulfate.
6 Slowly add the mixture to 300 ml cold acetone in an ice bath to precipitate the product.
7 Collect the white precipitate by vacuum filtration.
8 Check the acetone filtrate by HPLC for product. If there is a significant amount, concentrate the filtrate to a small volume, chill, and collect the additional product by vacuum filtration. Combine it with the rest.

### Purify 7‐methylguanosine

9 Suspend solid crude product in 300 ml of 95% ethanol, stir 5 min, and collect by filtration.
10 Suspend solid product in 300 ml ethyl ether, stir 5 min, and collect by filtration.
11 Dry pure product over P_2_O_5_ in a vacuum desiccator overnight.
  The yield is usually 80% to 90%. It is very important to keep this compound dry. It should be transferred to a bottle with a tight lid, the air should be displaced with nitrogen, and it should be stored at –20°C. It can be kept for up to 3 months.

## Basic Protocol 2 SYNTHESIS OF [2‐^13^C‐1,7,NH_2_‐^15^N_3_]‐ AND [8‐^13^C‐1,7,NH_2_‐^15^N_3_]GUANOSINE

**Figure 2:**
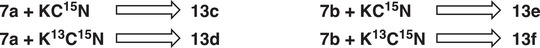
Reagent combinations needed to convert labeled adenosines **7a/b** to labeled guanosines **13c‐f**.

As shown in Figure 1, in the adenosine to guanosine transformation (Shallop & Jones, 2000; Zhao et al., 1997), the adenosine amino group becomes the guanosine N1, while the guanosine amino and C2 come from potassium cyanide. The first step is the oxidation of adenosine (**7a/b**) to the N1 oxide (**8a/b**), which is followed by a one‐flask set of reactions without purification to give **12c‐f**. Labeled cyanogen bromide is generated in situ from labeled potassium cyanide and bromine, and its reaction with **8a/b** gives the oxadiazolidines **9c‐f**. Treatment with triethylamine opens the oxadiazolidine ring, allowing the N1 oxide to be methylated by methyl iodide to give **10c‐f**. Aqueous sodium hydroxide then opens the pyrimidine ring, which allows rotation around the C5‐C6 bond of **11c‐f**. This rotation brings the cyano group near the deformylated 3‐amino group to facilitate ring closure upon neutralization and heating to give **12c‐f**. Enzymatic deamination then gives the final products, **13c‐f**. The reagent combinations to convert labeled adenosines to labeled guanosines are summarized in Figure 2.

### Materials

[7,NH_2_‐^15^N_2_]Adenosine (**7a**) or [8‐^13^C‐7,NH_2_‐^15^N_2_]adenosine (**7b**; Basic Protocol 1)
50% (v/v) methanol
3‐Chloroperoxybenzoic acid (MCPBA), purified (see recipe)
Ethyl ether
Phosphorous pentoxide (P_2_O_5_)
[^13^C,^15^N]Cyanogen bromide or [^15^N]cyanogen bromide, freshly prepared (see recipe)
0.1 M potassium phosphate (KH_2_PO_4_), pH 7.5
Dimethyl formamide (DMF), anhydrous
Acetonitrile
Triethylamine
Nitrogen gas source
Methyl iodide
0.1 M NaOH
1 M HCl
95% (v/v) ethanol
Adenosine deaminase (ADA; MilliporeSigma)

100‐ml round‐bottom flasks
Rotary evaporator
Vacuum desiccator
Oil bath (silicone oil), 60°C

Additional reagents and equipment for analytical and preparative reversed‐phase high‐performance liquid chromatography (HPLC; Current Protocols article: Sinha & Jung, 2015)

### Synthesize [7,NH_2_‐^15^N_2_]‐ and [8‐^13^C‐7,NH_2_‐^15^N_2_]adenosine‐N1‐oxide (8a/b)

1 Weigh 1.35 g (5.0 mmol) of [7,NH_2_‐^15^N_2_]adenosine (**7a**) or [8‐^13^C‐7,NH_2_‐^15^N_2_]adenosine (**7b**) into a 100‐ml round‐bottom flask. Add a stir bar and 50 ml of 50% methanol.
2 Add 1.72 g (10 mmol, 2 eq) purified MCPBA, cover the flask with aluminum foil, stir 3 to 4 hr, and monitor the reaction for completeness by HPLC.
  Any reversed‐phase column can be used.
3 Dilute solution with 25 ml water and wash with three 50‐ml portions of ethyl ether.
4 Use a rotary evaporator to concentrate the aqueous solution to a small volume, purify **8a/b** by preparative reversed‐phase HPLC, and dry it over P_2_O_5_ in a vacuum desiccator overnight in a 100‐ml round‐bottom flask.
  The yield is usually 90% to 95%. See Table 1 for various data on this and other intermediates and products.

### Synthesize labeled 2‐amino‐6‐(methoxyamino)‐9‐(β‐d‐ribofuranosyl)purines (12c‐f)

5 Dissolve 1.43 g (5.0 mmol) **8a/b** in 40 ml water.
6 Add 7.5 mmol freshly prepared [^13^C,^15^N]cyanogen bromide or [^15^N]cyanogen bromide, as appropriate for the desired labeling pattern, stir 2 hr, and monitor reaction for completeness by HPLC.
  CAUTION: The waste in the evaporator trap may contain excess cyanogen bromide. Dispose of this waste in an appropriately designated area, following the guidelines provided by the local safety officer.
7 Concentrate solution to a very small volume using a rotary evaporator, add 10 ml anhydrous DMF and 10 ml acetonitrile, and concentrate again. Repeat this drying process two more times.
  CAUTION: Be very careful to dispose of the waste from the evaporator trap according to accepted regulations because it may contain excess cyanogen bromide.
8 Add 25 ml anhydrous DMF and 2.8 ml (20 mmol, 4 eq) triethylamine under nitrogen gas.
9 Stir 45 min and then slowly add 2.5 ml (40 mmol, 8 eq) methyl iodide.
  Do not wait >1 hr to add methyl iodide.
10 Cover flask with aluminum foil and stir 3 to 4 hr while monitoring the reaction for completeness by HPLC.
11 Concentrate solution to a yellow oil and add 85 ml of 0.1 M NaOH.
  Do not wait >4 hr to concentrate the solution and add the NaOH.
  CAUTION: The waste from the evaporator trap must be carefully disposed, according to accepted regulations, because it may contain excess methyl iodide.
12 Stir 20 min and then adjust pH to 7.4 with 1 M HCl.
13 Add 80 ml of 95% ethanol, attach a condenser, and heat solution in an oil bath at 60°C for 4 hr while monitoring the reaction for completeness by HPLC.
14 Concentrate solution to a small volume using a rotary evaporator, purify **12c‐f** by reversed‐phase HPLC, and dry over P_2_O_5_ in a vacuum desiccator overnight in a 100‐ml round‐bottom flask.
  The yield is usually 80% to 90%.

### Synthesize labeled guanosines (13c‐f)

15 Dissolve 1.58 g (5 mmol) **12c‐f** in 80 ml of 0.1 M KH_2_PO_4_, pH 7.5.
16 Add 300 units of adenosine deaminase, stopper the flask, and heat 4 days at 37°C with gentle agitation.
  Most of the product should crystallize out during this time.
17 Cool mixture in an ice bath and collect crude **13c‐f** by filtration.
18 Purify **13c‐f** by recrystallization from water and dry it over P_2_O_5_ in a vacuum desiccator overnight.
  The yield is usually 75% to 85%. See Tables 2 and 3 for NMR chemical shifts and coupling constants.

## REAGENTS AND SOLUTIONS

### 3‐Chloroperoxybenzoic acid (MCPBA), purified

Dissolve 10 g MCPBA in 200 ml ether and wash with three 150‐ml portions of 0.1 M aqueous potassium phosphate, pH 7.5. Concentrate to dryness and dry over P_2_O_5_ in a vacuum desiccator overnight.
Store up to 3 months at –20°C in a bottle with a tight cap.

CAUTION: MCPBA is potentially explosive and must be handled with care. Consult the technical data sheet: https://static.fishersci.eu/content/dam/fishersci/en_EU/suppliers/scs/M‐CPBA_data_sheet.pdf

Commercial MCPBA is contaminated with 40% to 50% 3‐chlorobenzoic acid, which can be removed by extraction.

### [^13^C,^15^N]Cyanogen bromide

Place 3 ml water into a small pear‐shaped flask and add 1.2 g (0.38 ml, 7.5 mmol) bromine. Chill the flask in an ice bath, slowly add 0.50 g (7.5 mmol, 1 eq) [^13^C,^15^N]potassium cyanide dissolved in 20 ml water, and stir 30 min. Draw the solution into a syringe in order to add it to a reaction.

CAUTION: Use great care in handling bromine because it is highly toxic, is volatile, and can cause severe burns. Place a balance in a hood, draw up the required volume of bromine into a preweighed syringe, weigh the syringe, and adjust accordingly. Potassium cyanide and cyanogen bromide are also highly toxic and must be handled with great care and appropriate lab safety practices (i.e., use of fumehood and personal protective equipment).

### [^15^N]Cyanogen bromide

Prepare as for [^13^C,^15^N]cyanogen bromide (see recipe) but use [^15^N]potassium cyanide instead of [^13^C,^15^N]potassium cyanide.

### [^13^C]Sodium ethyl xanthate ([^13^C]NaSCSOEt)

Dissolve 0.40 g (10 mmol) NaOH in 40 ml absolute ethanol. Add 0.77 g (10 mmol, 1 eq) [^13^C]carbon disulfide ([^13^C]CS_2_; Isotec or Cambridge Isotope Laboratories). Cover the flask with aluminum foil and stir the solution overnight at room temperature. Concentrate to dryness and dry over P_2_O_5_ in a vacuum desiccator overnight. Store up to 6 months at –20°C.

The yield is usually quantitative.

## COMMENTARY

### Background Information

^15^N NMR studies of specifically ^15^N‐labeled DNA and RNA fragments have provided significant information about local interactions at nitrogen atoms, such as hydrogen bonding, stacking, and protonation (Wang, Gao, Gaffney, & Jones, 1991; Zhang, Gaffney, & Jones, 1997, 1998). The use of one or more multi‐labeled nucleosides can provide more information than single‐labeled nucleosides but only as long as all signals can be distinguished. The use of ^13^C tags adjacent to different nitrogens in a pair of nucleosides was designed to allow such differentiation (Abad, Shallop, Gaffney, & Jones, 1998; Shallop & Jones, 2000; Zhao et al., 1997). In addition, the ^13^C chemical shifts can provide valuable information. Recent work using these labeled DNA or RNA fragments include Dayie, Olenginski, and Taiwo (2022); Olenginski, Taiwo, Leblanc, and Dayie (2021); Olengiinsky (2021); Goldberga et al. (2019); Becette, Olenginski, and Dayie (2019); Nussbaumer et al. (2017); Dallmann et al. (2016); and Lagoya (2002).

The route to labeled adenosine described here (Pagano et al., 1995) starts with a direct nitrosation, which is more convenient than the azo coupling described earlier (Gaffney, Kung, & Jones, 1990). This route has been designed so as to remove both thiol groups (in the case of **3b**) in the same step. The enzymatic coupling is done with 6‐chloropurine because the subsequent displacement with [^15^N]NH_3_ can be done using much milder conditions. Also, the lipophilicity of the 6‐chloro group helps in the purification of the nucleoside. The purine nucleoside phosphorylase uses 7‐methylguanosine as a sugar donor and both generates the ribose‐α‐1‐phosphate and couples it to the 6‐chloropurine. The reaction is driven to completion by precipitation from solution of the very insoluble 7‐methylguanine. The final amination has been optimized so that it only requires two equivalents of [^15^N]NH_4_Cl with KHCO_3_. Although not described here, [7,NH_2_‐^15^N_2_]adenosine can be converted to [1,7,NH_2_‐^15^N_3_]adenosine (Pagano, Zhao, Shallop, & Jones, 1998). Deamination of any of the adenosines to the corresponding labeled inosine is easily effected. In addition, synthesis of the labeled deoxynucleosides using purine nucleoside phosphorylase (PNP) and 7‐methyldeoxyguanosine is straightforward (Drenichev et al., 2022).

The adenosine to guanosine transformation described here (Shallop & Jones, 2000; Zhao et al., 1997) is based on a previous method (Goswami & Jones, 1991) that was in turn derived from earlier work (Ueda, Miura, & Kasai, 1978).

### Critical Parameters

In the chlorination that converts **4a/b** to **5a/b**, it is essential to remove all excess POCl_3_, or heat generated during subsequent neutralization with ammonia will cause the reaction to reverse to some extent. During the one‐flask set of reactions for the adenosine to guanosine transformation, the intermediates are not particularly stable, and the reactions should not be left for longer than the stated times. After formation of cyanogen bromide, there should be no excess bromine present (as indicated by no orange color present). The order of addition during the formation of cyanogen bromide is also important. In the final enzymatic deamination to make **13c‐f**, it is important that **12c‐f** be purified for the enzymatic reaction to work satisfactorily.

### Understanding Results

The early steps in these protocols generally give high yields, even for inexperienced workers. The later steps are more challenging, and, with experience, the stated yields can be obtained. Because most of these compounds are fairly polar, the use of TLC to monitor reactions is not very convenient. Reversed‐phase HPLC is much more informative, with UV λ_max_ data from an HPLC system with a diode array detector and mass data from an LCMS being particularly helpful.

### Time Considerations

The total time for Basic Protocol 1 is 2 to 3 weeks and that for Basic Protocol 2 is 1 to 2 weeks. The Support Protocol requires 1 to 2 days.

### Author Contributions

**Roger A. Jones**: Conceptualization, project administration, supervision, writing original draft, writing review and editing; **Barbara L. Gaffney**: Investigation, project administration, supervision, writing original draft, writing review and editing.

### Conflict of Interest

The authors declare no conflict of interest.

## Data Availability

Data sharing not applicable – no new data generated.
